# 17β-Estradiol supplementation changes gut microbiota diversity in intact and colorectal cancer-induced ICR male mice

**DOI:** 10.1038/s41598-020-69112-w

**Published:** 2020-07-23

**Authors:** Chin-Hee Song, Nayoung Kim, Ryoung Hee Nam, Soo In Choi, Ha-Na Lee, Young-Joon Surh

**Affiliations:** 1 Department of Internal Medicine, Seoul National University Bundang Hospital, Seongnam, Gyeonggi-do South Korea; 2 Department of Internal Medicine and Liver Research Institute, Seoul National University College of Medicine, Seoul, South Korea; 3 Laboratory of Immunology, Division of Biotechnology Review and Research-III, Office of Biotechnology Products, Center for Drug Evaluation and Research, Food and Drug Administration, Silver Spring, MD 20993 USA; 4 Tumor Microenvironment Global Core Research Center, Seoul National University College of Pharmacy, Seoul, South Korea

**Keywords:** Cancer, Microbiology

## Abstract

The composition of the gut microbiota is influenced by sex hormones and colorectal cancer (CRC). Previously, we reported that 17β-estradiol (E2) inhibits azoxymethane/dextran sulfate sodium (AOM/DSS)-induced tumorigenesis in male mice. Here, we investigated whether the composition of the gut microbiota is different between male and female, and is regulated by estrogen as a secondary outcome of previous studies. We established four groups of mice based on the sex and estrogen status [ovariectomized (OVX) female and E2-treated male]. Additionally, three groups of males were established by treating them with AOM/DSS, and E2, after subjecting them to AOM/DSS treatment. The mice were sacrificed at 21 weeks old. The composition of the gut microbiota was analyzed using 16S rRNA metagenomics sequencing. We observed a significant increase in the microbial diversity (Chao1 index) in females, males supplemented with E2, and males treated with AOM/DSS/E2 compared with normal males. In normal physiological condition, sex difference and E2 treatment did not affect the ratio of Firmicutes/Bacteroidetes (F/B). However, in AOM/DSS-treated male mice, E2 supplementation showed significantly lower level of the F/B ratio. The ratio of commensal bacteria to opportunistic pathogens was higher in females and E2-treated males compared to normal males and females subjected to OVX. Unexpectedly, this ratio was higher in the AOM/DSS group than that determined in other males and the AOM/DSS/E2 group. Our findings suggest that estrogen alters the gut microbiota in ICR (CrljOri:CD1) mice, particularly AOM/DSS-treated males, by decreasing the F/B ratio and changing Shannon and Simpson index by supply of estrogen. This highlights another possibility that estrogen could cause changes in the gut microbiota, thereby reducing the risk of developing CRC.

## Introduction

Colorectal cancer (CRC) is the third leading cause of cancer death in both men and women in the United States, with an estimation of 101,420 and 51,020 new cases of cancer and cancer-related deaths, respectively, in 2019^1^. Chronic inflammation following viral or bacterial infections is considered as an important risk factor involved in human carcinogenesis, including CRC^2^ and its association has been reported, particularly in infections such as viral hepatitis and hepatocellular carcinoma^3^, as well as that of *Helicobacter pylori* and gastric adenocarcinoma^4^. Moreover, it has been reported that opportunistic pathogens play a role in colorectal carcinogenesis. *Fusobacterium nucleatum*, *Escherichia coli*, *Streptococcus gallolyticus* (formerly known as *S. bovis*), *Enterococcus faecalis*, and enterotoxigenic *Bacteroides fragilis* (a toxin-producing bacteria) have been considered as candidate microorganisms responsible for triggering the colorectal carcinogenesis^5^. Additionally, results obtained from several animal experiments have suggested that these bacteria could be the causative microorganisms responsible for colorectal carcinogenesis^5^. According to a recent report, infection with *F. nucleatum* had promoted chemoresistance to 5-fluorouracil by upregulating the expression of baculoviral IAP repeat-containing 3 (BIRC3) in CRC cells^6^. Additionally, abundance of *F. nucleatum* could be correlated with the chemoresistance observed in patients with advanced CRC^6^. However, a clear cause-and-effect relationship between *F. nucleatum* and CRC has not been established.

The composition of the gut microbiota is driven by a number of factors such as diet, antibiotic therapy, maternal microbiota, and genotype of the individual^7–9^. Thus, conducting research on the gut microbiota of humans is very difficult. Instead, genetically homogenous mice housed and administered with identical controlled environments and diets, respectively, render lesser variability in conditions. A mouse model developed by treating the animals with azoxymethane (AOM, a pro-carcinogenic agent) and dextran sulfate sodium (DSS, a chemical colitogen) is most widely used in the investigation of the molecular pathogenesis of colitis and colitis-associated CRC^10,11^. This animal model is well established for the identification of multistep tumor progression based on the presence of aberrant crypt foci (ACF)-adenoma-carcinoma sequence, through which, molecular alterations are assessed in specific phases of the carcinogenic process^12^. According to several reports, the richness and diversity of the composition of the gut microbiota change upon treatment with AOM and/or DSS^13–15^. Particularly, in female C57BL/6 mice treated with 4% DSS, the relative abundance of the *Lactobacillus* group was lower while that of Enterobacteriaceae, *Akkermansia* and *Desulfovibrio* were higher^16^.

It is well known that the occurrence of colorectal tumors is more frequent in men than women^17^, and differences in the incidence of CRC, which are associated with sex, exist worldwide^18^. Many epidemiological data have suggested that the female sex hormone estrogen, exerts protective effects against CRC development^19,20^. Therefore, in postmenopausal women, the chances of CRC incidence may increase. Indeed, CRC is considered as a major cause of death in elderly women above the age of 65 years in Korea^21^. Interestingly, emerging evidence has suggested that the interaction between estrogen and gut microbiota is likely to affect the microenvironment^22^ and metabolism of the host^23^. It has been revealed that the gut microbiota is regulated by estrogen, and the levels of estrogens are also significantly affected by gut microbiota composition^24^. In animal models, bilateral ovariectomy (OVX) causes microbial dysbiosis^25,26^. Furthermore, short-chain fatty acids (SCFA) produced by the gut microbiota are recognized as metabolic signaling molecules and their levels are reported to be significantly decreased in OVX rats^26^. It has been reported that, in humans, OVX is associated with an increment in the relative abundance of *Clostridium bolteae*^27^. In men and postmenopausal women, the levels of total urinary estrogens could be strongly correlated with the species richness and α-diversity of the gut microbiota^28^. These non-ovarian systemic estrogens were positively correlated with the taxonomic abundance of Clostridia, including non-Clostridiales, and three genera belonging to the Ruminococcaceae family^28^. Additionally, alteration in the composition of the gut microbiota including an increase in the levels of Bifidobacteria and suppression of Clostridiaceae was observed in postmenopausal women upon administration of phytoestrogens such as soy isoflavone, which are plant-derived compounds demonstrating estrogenic activities^29^.

Previously, we have reported that the severity of CRC in the AOM/DSS murine model was greater in male mice than female mice, as evidenced by the increased levels of inflammatory mediators such as MPO and IL-1β^30^. E2 (10 mg/kg) was found to suppress the induction of CRC by upregulating nuclear factor-erythroid 2-related factor 2 (Nrf2) in male ICR mice treated with AOM/DSS^31^. Furthermore, E2 treatement significantly downregulated the expressions of COX-2 or iNOS in TNF-α-stimulated human female normal epithelial CCD841CoN cells^32^ and mouse embryonic fibroblasts (MEFs), collectively indicating its anti-inflammatory effect^33^.

Based on this, we hypothesized that sex hormones could affect the gut microbiota and subsequent development of CRC. To evaluate this hypothesis, we focused on the microbial alterations, particularly the relative abundance of opportunistic pathogens, in normal or AOM/DSS-treated male mice treated with estrogen, and in female mice eliminated the presence of estrogen through ovariectomy, and investigated their associations with each other.

## Results

### Effect of sex and CRC on clustering and phylum compositions following supplementation with estrogen

**Figure 1:**
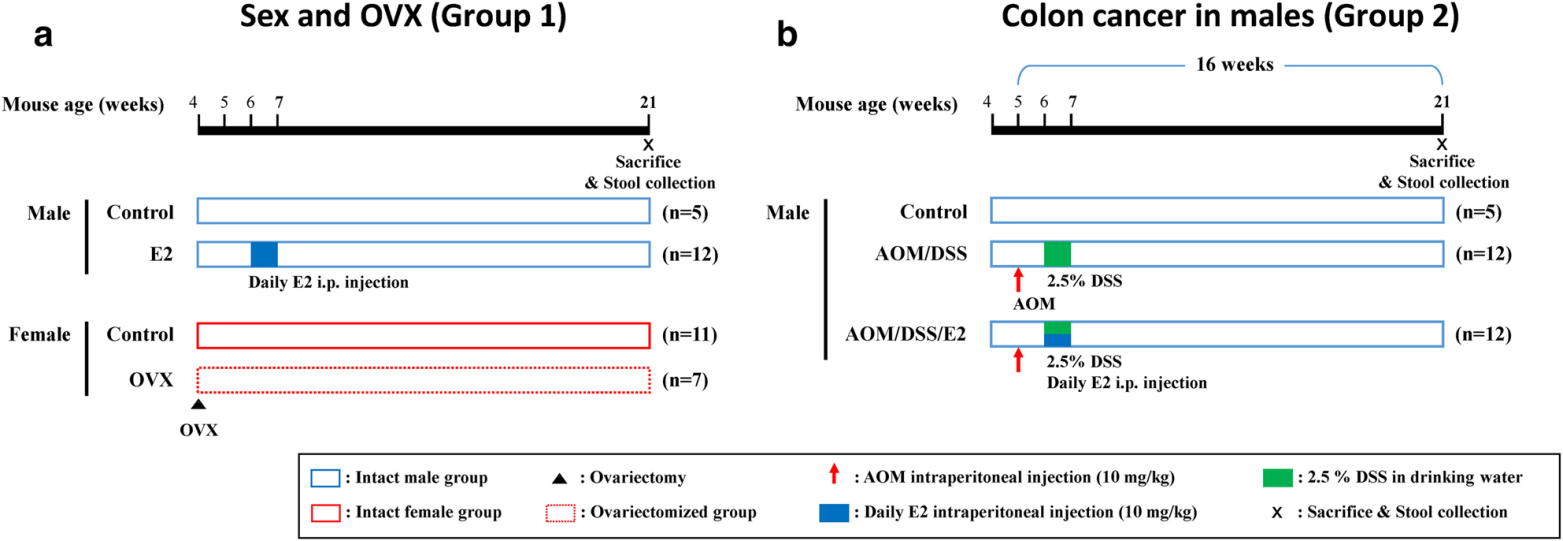
Experimental scheme for evaluating the roles of sex and CRC following E2 on gut microbial changes. (**a**) Group 1 samples: Male_Con, Male_E2, Female_Con, and Female_OVX. Ovariectomy operation was performed at 4 weeks aged female mice. E2 was supplemented by intraperitoneal injection for one week at 6 weeks aged male mice. (**b**) Group 2 samples: Male_Con, Male_AOM/DSS, and Male_AOM/DSS/E2. At weeks 5, male mice were injected with AOM and were provided DSS in drinking water (2.5%) and E2 supplementation for 1 week at weeks 6. Fecal samples were collected at weeks 21.

The experimental groups were distinguished into two categories, which were as follows: sex and OVX as Group 1, and colon cancer in males as Group 2. Group 1 was further divided into 4 subgroups, which were as follows: normal male control mice (n = 5), E2 supplemented male mice (n = 12), normal female control mice (n = 11), and ovariectomized (OVX) female mice (n = 7) (Fig. 1a). Similarly, group 2 was divided into 3 subgroups, which were as follows: normal male control mice (n = 5), AOM/DSS-treated male mice (n = 12), and AOM/DSS-treated male mice supplemented with E2 (n = 12) (Fig. 1b). Female mice were subjected to a sham surgery or ovariectomy at 4-week-old. To induce colitis-associated CRC, 5-week-old male mice were injected intraperitoneally with 10 mg/kg AOM and were given 2.5% DSS in the drinking water for 7 days starting 1 week following the injection of AOM. Estrogen supplemented groups were received a daily intraperitoneal injection of E2 for 7 days, and other groups were received olive oil as a vehicle (Fig. 1). Their feces were freshly collected and immediately frozen in liquid nitrogen.

**Figure 2:**
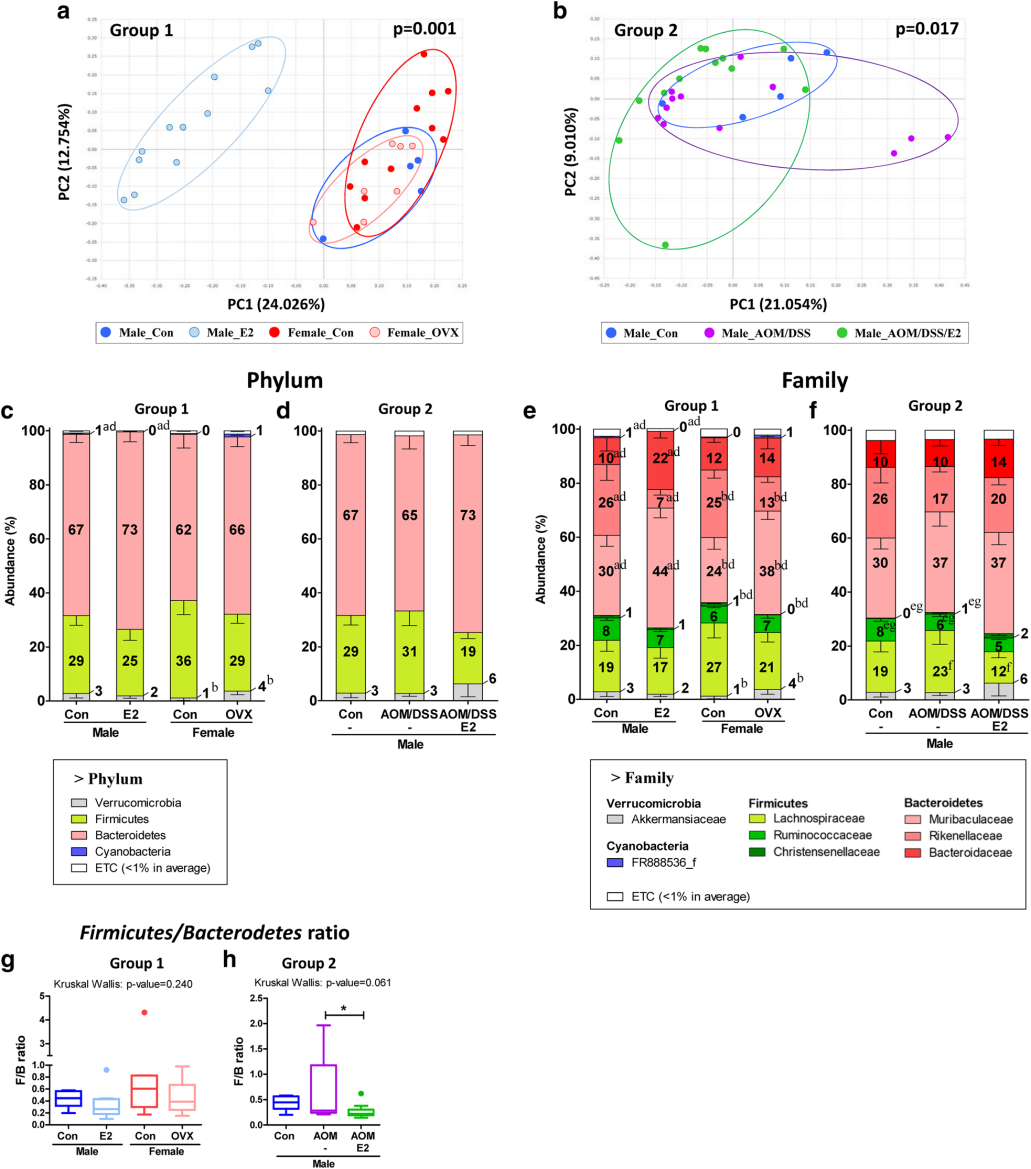
Sample clustering and gut microbiota compositions at the Phylum and Family levels. Metagenome sequencing was performed with 16S rRNA from mouse fecal samples. Clustering of (**a**) Group 1 and (**b**) Group 2 mice samples by principal coordinates analysis (PCoA) at the species level. PERMANOVA test for the dissimilarity of bacterial population structures was performed in (**a**) and (**b**). Taxonomic composition at the (**c**, **d**) phylum and (**e**, **f**) family level. Microbial composition of fecal contents from (**c**, **e**) Group 1 and (**d**, **f**) Group 2. Mann–Whitney U-test for comparison difference between independent two groups was performed in (**a**), (**b**), (**c**), (**e**), and (**f**). The difference between Group1 and between Group 2 was analysed by Kruskal–Wallis H test in d and g. (**a**), *p* < 0.05 between Male_Con vs. Male_E2; (**b**), *p* < 0.05 between Female_Con vs. Female_OVX; (**c**), *p* < 0.05 between Male_Con vs. Female_Con; (**d**), *p* < 0.05 between Group1; (**e**), *p* < 0.05 between Male_Con vs. Male_AOM/DSS; (**f**), *p* < 0.05 between Male_AOM/DSS vs. Male_AOM/DSS/E2; (**g**), *p* < 0.05 between Group2 (Supplementary Table 1, 2). Firmicutes/Bacteroidetes ratio of (**g**) Group 1 and (**h**) Group 2 mice. *, *p* < 0.05 for comparison between Male_AOM/DSS vs. Male_AOM/DSS/E2. Data are expressed as the mean ± SEM. Whiskers show the minimum and maximum values. Con, control; E2, 17β-Estradiol; OVX, ovariectomized; AOM, azoxymethane; DSS, dextran sulfate sodium salt.

After genomic DNA preparation from stools, high-throughput 16S rRNA metagenome sequencing was carried out to determine whether differences in the gut microbiome are influenced by sex of the individual and treatment with E2 with or without AOM/DSS treatment in male ICR mice. The rarefaction curve of all 59 samples formed a plateau (Supplementary Fig. 1). Variations occurring in the gut microbial communities among different groups were assessed based on their generalized UniFrac distances. Results of the principal coordinates analysis (PCoA) showed that the bacterial communities could be distinguished based on supplementation with estrogen (Fig. 2a, Supplementary Fig. 2a). Except the Male_E2 samples belonging to Group 1, which were representative of the sex and OVX groups, no clear divergence based on the differences in sex or OVX and AOM/DSS or estrogen treatments was observed in samples obtained from members of Group 1 and Group 2, which is indicative of the development of colon cancer in males, respectively (Fig. 2a,b, Supplementary Fig. 2a, b). PERMANOVA results demonstrated the beta set-significance between Group 1 (*p* = 0.001) and between Group 2 (*p* = 0.017) (Fig. 2a,b).

In Group 1, different compositions of the gut microbiota at the levels of both phylum and family taxa were observed (Fig. 2c,e, Supplementary Table 1, 2). At the phylum level, the abundance ratio of Cyanobacteria decreased significantly by estrogen supplementation in the groups involving male members (*p* < 0.001 based on the Kruskal–Wallis test; *p* < 0.001 for Male_Con vs. Male_E2) (Fig. 2c, Supplementary Table 1). Furthermore, the abundance ratio of Verrucomicrobia increased significantly in the absence of estrogen (OVX) in the groups with females (*p* = 0.054 based on the Kruskal–Wallis test; *p* = 0.007 for Female_con vs. Female_OVX) (Fig. 2c, Supplementary Table 1). Differences in the gut microbiota of members belonging to Group 2 were observed only at the family taxa level (Fig. 2d,f, Supplementary Table 1, 2). Furthermore, the ratio of Firmicutes to Bacteroidetes (F/B ratio) did not differ significantly among the members of Group 1 (Fig. 2g). Interestingly, the F/B ratio was significantly lower in the AOM/DSS group supplemented with E2 than that of their AOM/DSS counterparts in Group 2 (*p* = 0.061 for the Kruskal–Wallis test, *p* = 0.050 for Male_AOM/DSS vs Male_AOM/DSS/E2) (Fig. 2h).

The counts and abundance ratios of all operational taxonomic units (OTU) are presented in Supplementary Dataset 1 and 2. All *p*-values and q-values of the false discovery rate (FDR) obtained by comparing the results of Group 1 and Group 2 are shown in Supplementary Dataset 3. The result of LDA effect size obtained by comparing between Group 1 and between Group 2 are shown in Supplementary Dataset 4. The microbial compositions at the genus levels are presented in Supplementary Fig. 3a-b. Members of the genus *Alistipes* were the most dominant organisms detected in the male and female control groups, accounting, for 26% and 24% of the total, respectively (Supplementary Fig. 3a). In the estrogen supplemented male (Male_E2) and estrogen eliminated female (Female_OVX) groups, *Bacteroides* was the most prominent genus identified, accounting for nearly 21% and 14% of the total, respectively (Supplementary Fig. 3a). In Group 2, *Alistipes* decreased in the Male_AOM/DSS group (17%) compared to Male_Con group (26%). Interestingly, the decrement of *Alistipes* was slightly recovered by estrogen supplementation (Male_AOM/DSS/E2) (20%) in Male_AOM/DSS group (Supplementary Fig. 3b).

### Influence of sex and CRC following estrogen supplementation on the diversity of the gut microbiota

**Figure 3:**
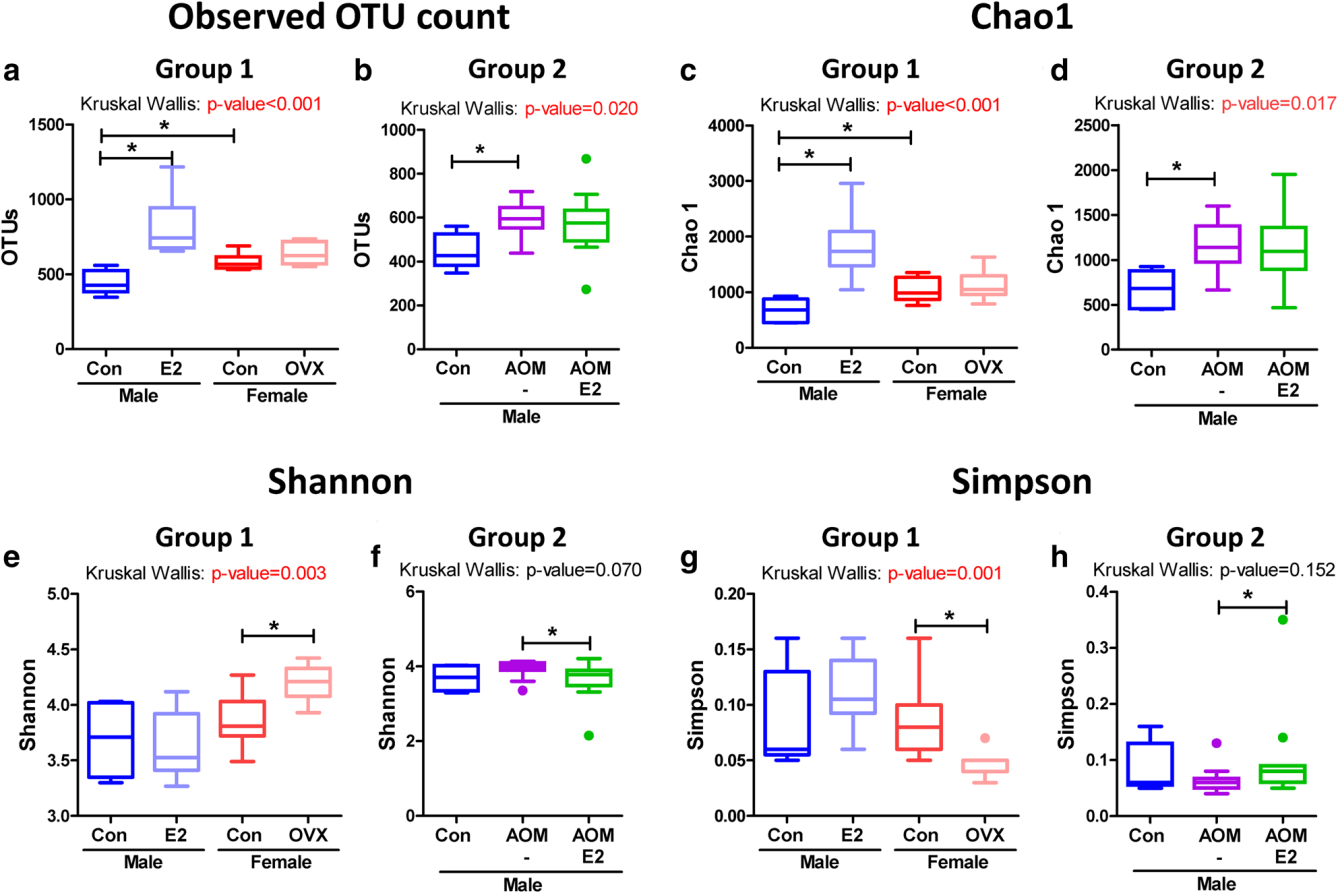
Alpha diversity of gut microbiota. (**a**,**b**) Observed OTU count, (**c**,**d**) species richness (Chao1), (**e**,**f**) alpha diversity (Shannon), and (**g**, **h**) alpha diversity (Simpson) of the gut microbiomes of (**a**,**c**,**e**, **g**) Group 1 and (**b**,**d**,**f**,**h**) Group 2 mice. Data are expressed as the mean ± SEM. Whiskers show the minimum and maximum values. *p*-values to Kruskal–Wallis test is designated on the figure; ^∗^, *p* < 0.05 according to Mann–Whitney U-test. Con, control; E2, 17β-Estradiol; OVX, ovariectomized; AOM, azoxymethane; DSS, dextran sulfate sodium salt.

It was observed that, in Group 1, the OTU count increased significantly in both, estrogen supplemented male and female control groups, compared to that observed in the male control group (*p* = 0.002 for Male_Con vs. Male_E2, *p* = 0.008 for Male_Con vs. Female_Con) (Fig. 3a, Table 1). Similar to the OTU count, species richness of the gut microbiota, as indicated by the Chao 1 index, was also found to be significantly increased in estrogen supplemented male and female control groups compared to that of the male control (*p* = 0.002 for Male_Con vs. Male_E2, *p* = 0.008 for Male_Con vs. Female_Con) (Fig. 3c, Table 1). However, the observed OTU count and Chao 1 index did not vary post ovariectomy compared to those of the female control group (Fig. 3a,c, Table 1). Interestingly, significant alterations in the alpha diversity indices were observed in the OVX group compared to that of the female control (Shannon index, *p* = 0.013; Simpson index, *p* = 0.006 for Female_Con vs. Female_OVX) (Fig. 3e,g, Table 1). We observed that, in Group 2, treatment of male mice with AOM/DSS resulted in significant alteration in the OTU count (*p* = 0.006 for Male_Con vs. Male_AOM/DSS) and values of the Chao 1 index compared to those determined in the male control group (Fig. 3b,d, Table 1). On the other hand, the alpha diversity indices (Shannon and Simpson) was significantly changed upon supplementation with E2 in the AOM/DSS male group (Shannon index, *p* = 0.033 and Simpson index, *p* = 0.028 for Male_AOM/DSS vs Male_AOM/DSS/E2) (Fig. 3f,h, Table 1).

**Table 1:** Alpha diversity of microbiota from fecal contents.

Group	No. of OTUs	Good’s library coverage (%)	Alpha-diversity				
			ACE	Chao1	Jackknife	Shannon	Simpson
**Group1**							
Male_con. (n = 5)	448	97.50	845.92	671.14	845.70	3.69	0.09
Male_E2 (n = 12)	809	93.32	2,979.60	1835.93	2,431.81	3.64	0.11
Female_con (n = 11)	588	96.12	1,296.39	1,042.18	1,332.23	3.87	0.08
Female_ovx (n = 7)	642	95.59	1554.89	1,139.11	1,347.69	4.20	0.05
*p*-value^a^	**0.002**	**0.002**	**0.002**	**0.002**	**0.002**	0.874	0.165
*p*-value^b^	0.094	0.221	0.298	0.342	0.964	**0.013**	**0.003**
*p*-value^c^	**0.008**	**0.020**	0.100	**0.008**	0.062	0.307	0.863
*p*-value^d^	** < 0.001**	** < 0.001**	** < 0.001**	** < 0.001**	** < 0.001**	**0.003**	**0.001**
**Group2**							
Male_con (n = 5)	448	97.50	845.92	671.14	845.70	3.69	0.09
Male_AOM/DSS (n = 12)	595	95.71	1706.74	1,150.79	1,367.99	3.94	0.06
Male_AOM/DSS/E2 (n = 12)	567	95.82	1639.04	1,133.03	1,440.71	3.64	0.10
*p*-value^e^	**0.006**	**0.006**	**0.008**	**0.006**	**0.020**	0.126	0.275
*p*-value^f^	0.386	0.644	0.603	0.773	0.773	**0.030**	0.055
*p*-value^g^	**0.020**	**0.018**	**0.026**	**0.017**	0.060	0.070	0.152

Data are presented as the medians. p-value < 0.05 was considered to be significant and was presented as boldface. Mann–Whitney U-test for comparison difference between independent two groups was performed in a, b, c, e, and f. The difference between Group1 and between Group2 was analysed by Kruskal–Wallis H test in d and g.

^a^*p*-values between Male_Con vs. Male_E2; ^b^*p*-values between Female_Con vs. Female_OVX; ^c^*p*-values between Male_Con vs. Female_Con; ^d^*p*-values between Group1; ^e^*p*-values between Male_Con vs. Male_AOM/DSS; ^f^*p*-values between Male_AOM/DSS vs. Male_AOM/DSS/E2; ^g^*p*-values between Group2. OTU, operational taxonomic unit; Con, control; E2, 17β-Estradiol; OVX, ovariectomized; AOM, azoxymethane; DSS, dextran sulfate sodium salt.

### Influence of sex and CRC due to estrogen supplementation on the composition of the gut microbiota

**Figure 4:**
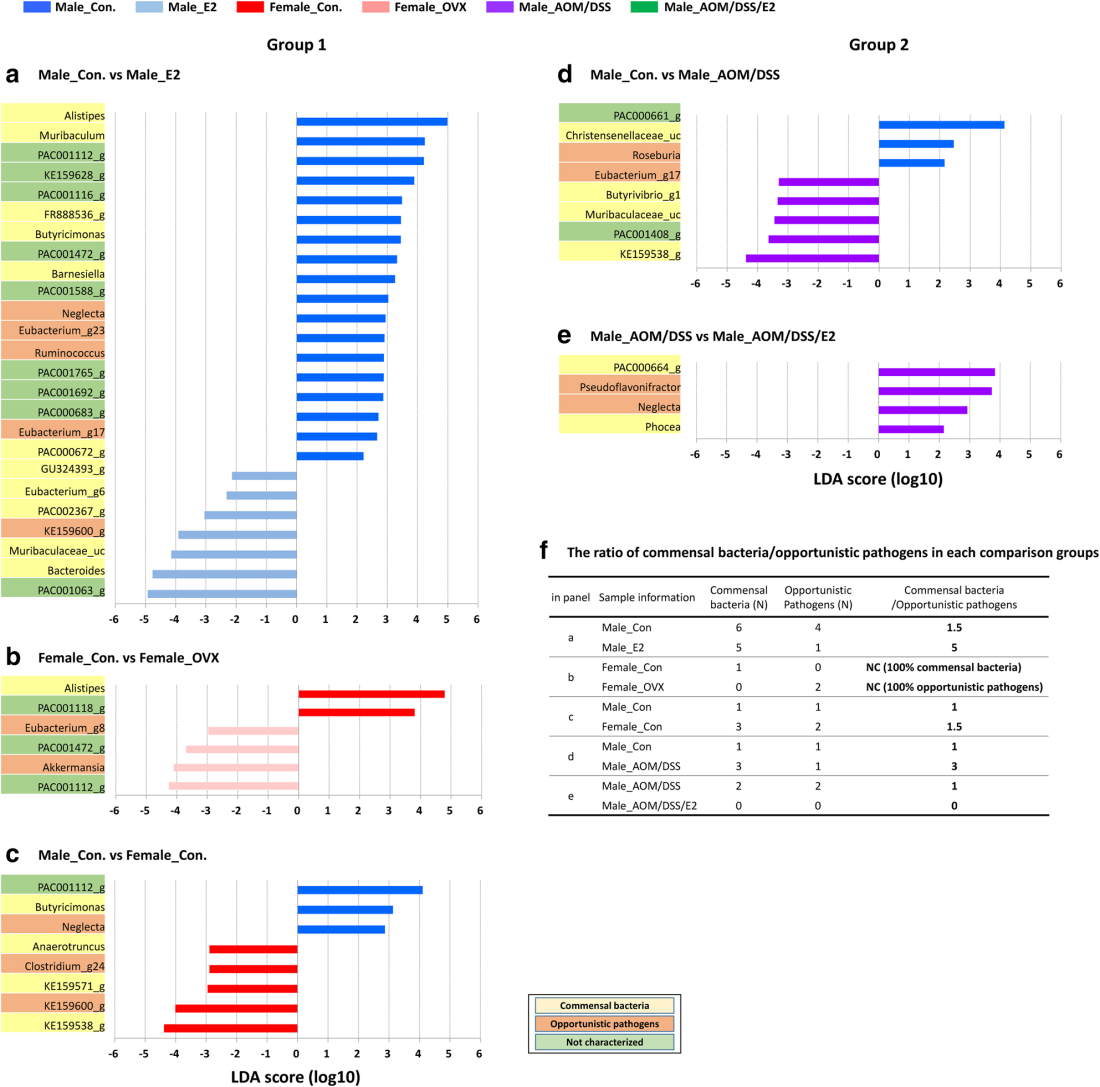
Alterations in the abundance ratio of the gut microbiota by estrogen on sex and CRC: linear discriminant analysis (LDA) effect size (LEfSe) analysis. Bar plots were generated by following process. (1) an alpha value for the factorial Kruskal–Wallis H test between assigned taxa compared to that of the groups with less than 0.05, (2) an alpha value for the pairwise Wilcoxon test among the taxonomic compositions of less than 0.05, (3) a threshold of the logarithmic LDA score for discriminative features less than 2.0, and (4) a multi-class analysis set as all-against-all. After this primary process, (5) overlapped bacteria selection, (6) search and classify the bacterial characteristics with reference to reported papers: commensal bacteria, opportunistic pathogens, not characterized, and (7) elimination of non-overlapped bacteria only at ‘not characterized’ bacteria. In LEfSe plot, all of commensal bacteria and opportunistic pathogens and only overlapped ‘not characterized’ bacteria are included. Bar plots at the genus level with significant differences in abundance with LEfSe analysis in (**a**–**c**) Group 1 (**d**,**e**) Group 2 mice. The color bars show the LDA scores of genes that enriched in indicated condition; (**a**, **c**, **d**) blue (male control mice), (**a**) sky blue (E2-treated male mice), (**b**, **c**) red (female control mice), (**b**) pink (ovariectomized female mice), and (**d**,**e**) purple (AOM/DSS-induced CRC). Each color on the genus name indicates the characteristics of each genus: yellow for commensal bacteria, orange for opportunistic pathogens, and green for not characterized bacteria. *p*-values were determined with the non-parametric factorial Kruskal–Wallis sum-rank test. (**f**) The ratio of commensal bacteria to opportunistic pathogens based on the LEfSe results in each comparison groups. Con, control; E2, 17β-Estradiol; OVX, ovariectomized; AOM, azoxymethane; DSS, dextran sulfate sodium salt; N, number; NC, not calculated.

We performed the linear discriminant analysis (LDA) effect size (LEfSe) analysis to identify the taxonomic biomarker in the gut microbiota. According to the results of the LEfSe analysis, the proportions of few genera constituting the gut microbiota were higher or lower significantly in both Group 1 and Group 2 (Fig. 4). In the male control mice, the relative abundances of opportunistic pathogens including *Neglecta, Eubacterium*_g23, *Ruminococcus*, and *Eubacterium*_g17 (*p* = 0.022, 0.039, 0.034, and 0.026, respectively) decreased in response to estrogen supplementation (Fig. 4a). In the female control mice, the relative abundances of opportunistic pathogens, including *Eubacterium*_g8 and *Akkermansia* (*p* = 0.022 and 0.007, respectively), increased while that of the commensal bacteria *Alistipes* (*p* = 0.013) decreased in response to the estrogen elimination (OVX) (Fig. 4b). In the male control mice, the relative abundance of the opportunistic pathogen *Neglecta* (*p* = 0.019) was higher and that of commensal bacteria including KE159571_g and KE159538_g (*p* = 0.041 and 0.006, respectively) was lower compared to that of the female control mice (Fig. 4c). Interestingly, abundance of the commensal bacteria *Butyricimonas* (*p* = 0.047) was lower in the female control mice compared to that observed in the males (Fig. 4c). In addition to these results, the ratio of commensal bacteria to opportunistic pathogens was found to be higher in Male_E2 and Female_Con groups than the Male_Con or Female_OVX groups (Fig. 4f).

The relative abundance of the opportunistic pathogen *Roseburia* (*p* = 0.009) decreased in the Male_AOM/DSS group compared to that in the Male_Con group (Fig. 4d). Surprisingly, the abundances of commensal bacteria including *Butyrivibrio*_g1, *Muribaculaceae*_uc, and KE159538_g (*p* = 0.022, 0.045 and 0.020, respectively) were higher in the Male_AOM/DSS group compared to that in the Male_Con group (Fig. 4d). Additionally, the abundances of all of commensal bacteria including PAC000664_g and *Phocea* (*p* = 0.007 and 0.001, respectively) and opportunistic pathogens including *Pseudoflavonifractor* and *Neglecta* (*p* = 0.012 and 0.003, respectively) diminished in the Male_AOM/DSS/E2 group compared to that in the Male_AOM/DSS group (Fig. 4e). Unexpectedly, the proportion of commensal bacteria to opportunistic pathogens was higher in the Male_AOM/DSS group than the Male_Con or Male_AOM/DSS/E2 groups (Fig. 4f).

### Enterotypes constituting the gut microbiota

**Figure 5:**
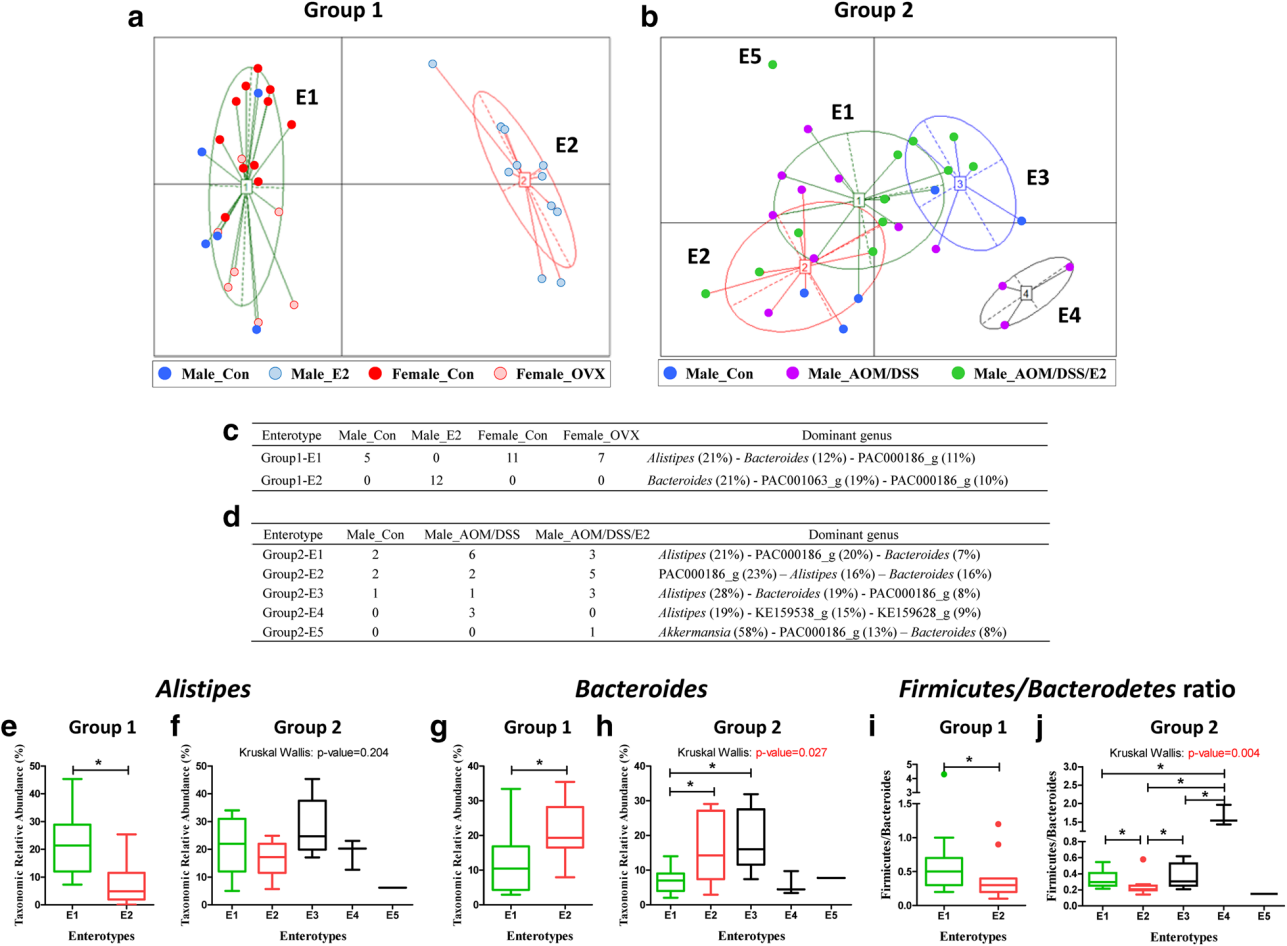
Enterotype clustering. (**a**) Two enterotypes (Group 1-E1, 2) of sex and OVX samples and (**b**) five enterotypes (Group 2-E1, 2, 3, 4, 5) of CRC male mice samples. The optimal cluster number was determined by maximizing the Calinski-Harabasz (CH) index value (Supplementary Fig. 4). The tables show the predominant genus in each enterotype of (**c**) Group 1 and (**d**) Group 2 mice samples. Relative abundance of (**e**, **f**) Alistipes and (**g**,**h**) Bacteroides, and (**i**,**j**) Firmicutes/Bacterodetes ratio of (**e**,**g**,**i**) Group 1 and (**f**,**h**,**j**) Group 2. Data are expressed as the mean ± SEM. Whiskers show the minimum and maximum values. *p*-values to Kruskal–Wallis test is designated on the figure; ^∗^, *p* < 0.05 according to Mann–Whitney U-test. Con, control; E2, 17β-Estradiol; OVX, ovariectomized; AOM, azoxymethane; DSS, dextran sulfate sodium salt.

To identify the properties of gut microbiota which could not be determined by the LEfSe analysis, Group 1 and Group 2 samples were further classified into enterotypes. The most optimal number ‘k’ was determined based on the highest value of the Calinski-Harabasz (CH) index (Supplementary Fig. 3). Samples constituting Group 1 were divided into two enterotypes because the highest CH value was 2 (Fig. 5a). Samples belonging to Group 2 were segregated into five enterotypes because the CH index was maximized when the cluster number was 5 (Fig. 5b). Enterotype 1 of Group 1 (Group 1-E1) was composed of 5 Male_Con., 11 Female_Con., and 7 Female_OVX samples, and it was dominated by the genus *Alistipes* (average 21%), followed by *Bacteroides* (12%), and PAC000186_g (11%) (Fig. 5c). Contrarily, enterotype 2 of Group 1 (Group 1-E2) comprised only of 12 Male_E2 group and was dominated by *Bacteroides* (21%), followed by PAC001063_g (19%), and PAC000186_g (10%) (Fig. 5c). Enterotype 1 of Group 2 (Group 2-E1) comprised of 2 Male_Con., 6 Male_AOM/DSS, and 3 Male_AOM/DSS/E2 samples (Fig. 5d). Group 2-E1 was dominated by *Alistipes* (21%), PAC000186_g (20%), and *Bacteroides* (7%) (Fig. 5d). Group 2-E2 comprised of 2 Male_Con., 2 Male_AOM/DSS, and 5 Male_AOM/DSS/E2 samples and was dominated by PAC000186_g (23%), *Alistipes* (16%), and *Bacteroides* (16%) (Fig. 5d). The Group 2-E3 comprised of 1 Male_Con., 1 Male_AOM/DSS, and 3 Male_AOM/DSS/E2 samples and was dominated by *Alistipes* (28%), *Bacteroides* (19%), and PAC000186_g (8%) (Fig. 5d). Group 2-E4 comprised of only 3 Male_AOM/DSS samples and was dominated by *Alistipes* (19%), KE159538_g (15%), and KE159628_g (9%) (Fig. 5d). Group 2-E5 comprised of only one Male_AOM/DSS/E2 sample and was dominated by *Akkermansia* (58%), PAC000186_g (13%), and KE159628_g (9%) (Fig. 5d). We further analyzed the relative abundance of the dominant genera between enterotypes within Group 1 and Group 2. In Group 1, the abundance of *Alistipes* (genus belonging to Bacteroidetes: Bacteroidia: Bacteroidales: Rikenellaceae) was significantly lower in enterotype 2 than that of enterotype 1 (*p* < 0.001) (Fig. 5e). The abundance of *Bacteroides* (genus belonging to Bacteroidetes: Bacteroidia: Bacteroidales: Bacteroidaceae) was significantly higher in enterotype 2 compared to enterotype 1 (*p* = 0.003) (Fig. 5g). The F/B ratio was also found to be significantly decreased in enterotype 2 compared to enterotype 1 (*p* = 0.045) (Fig. 5i). In Group 2, no significant difference on the abundance of *Alistipes* was observed (Fig. 5f). The abundance of *Bacteroides* were higher significantly in enterotypes 2 (*p* = 0.025) and 3 (*p* = 0.008) than that in enterotype 1 (*p* = 0.027 based on the Kruskal–Wallis test) (Fig. 5h). The F/B ratios were differently regulated in each enterotype (Fig. 5j).

### Functional profiles of microbial communities in mice of different sex and with CRC following estrogen supplementation

PICRUSt analysis was conducted to investigate the potential function of the gut microbiota and predict their classifications based on the Kyoto Encyclopedia of Genes and Genomes (KEGG) Ortholog (KO) (www.kegg.jp/kegg/kegg1.html). As a result, we identified differentially enriched functional biomarkers in Group 1 (Table 2) and Group 2 (Table 3). In Group 1, two orthologs were found to be higher in both Male_E2 and Female_Con. groups compared to those of the Male_Con. or Female_OVX groups, which were the ABC-2 type transport system ATP-binding protein (K01990) and TonB-dependent starch-binding outer membrane protein SusC (K21573) (Table 2). In Group 2, 17 orthologs increased and 8 orthologs decreased in Male_AOM/DSS group compared with those in the Male_Con. or Male_AOM/DSS/E2 groups, respectively (Table 3).

**Table 2:** Predicted functional biomarkers in Group 1.

In estrogen exposure group	Ortholog	Definition	LDA effect size	*p*-value	*p*-value (FDR)	Abundance ratio (mean, %)			
						Male_Con	Male_E2	Female_Con	Female_OVX
Increase	K21573	TonB-dependent starch-binding outer membrane protein SusC	1.6	0.024	0.039	0.140	0.340	0.227	0.219
Increase	K01990	ABC-2 type transport system ATP-binding protein	1.4	0.004	0.005	0.048	0.089	0.074	0.069

Data are presented as the medians. Data show the ortholog that increased by estrogen exposure (Male_E2 and Female_Con). LDA effect size, Linear discriminant analysis (LDA) effect size; FDR, false discovery rate; Con, control; E2, 17β-Estradiol; OVX, ovariectomized.

**Table 3:** Predicted functional biomarkers in Group 2.

In AOM/DSS group	Ortholog	Definition	LDA effect size	*p*-value	*p*-value (FDR)	Abundance ratio (mean, %)		
						Male_Con	Male_AOM/DSS	Male_AOM/DSS/E2
Increase	K06356	Phosphatase RapG regulator	2.1	0.041	0.048	0.175	0.201	0.125
Increase	K03200	Type IV secretion system protein VirB5	1.9	0.013	0.013	0.066	0.087	0.050
Increase	K02160	acetyl-CoA carboxylase biotin carboxyl carrier protein	1.8	0.038	0.044	0.059	0.078	0.047
Increase	K07655	Two-component system, OmpR family, sensor histidine kinase PrrB	1.8	0.036	0.042	0.040	0.054	0.028
Increase	K18298	Membrane fusion protein, multidrug efflux system	1.7	0.022	0.024	0.074	0.091	0.063
Increase	K02315	DNA replication protein DnaC	1.6	0.022	0.024	0.031	0.050	0.042
Increase	K07862	Serine/threonine transporter	1.5	0.039	0.046	0.053	0.059	0.044
Increase	K07447	Putative holliday junction resolvase	1.5	0.007	0.007	0.022	0.037	0.031
Increase	K05630	NEDD4-like E3 ubiquitin-protein ligase WWP2	1.5	0.019	0.020	0.055	0.060	0.045
Increase	K08095	Cutinase	1.4	0.022	0.024	0.021	0.029	0.016
Increase	K18037	Tyrosine-protein phosphatase non-receptor type 4	1.4	0.030	0.033	0.017	0.022	0.013
Increase	K07676	Two-component system, NarL family, sensor histidine kinase RcsD	1.3	0.012	0.013	0.033	0.037	0.021
Increase	K13212	RNA-binding protein Luc7-like 2	1.3	0.024	0.026	0.029	0.033	0.021
Increase	K16288	Dual serine/threonine and tyrosine protein kinase	1.3	0.022	0.025	0.015	0.021	0.011
Increase	K07440	Cholesterol 24-hydroxylase	1.3	0.025	0.027	0.054	0.058	0.038
Increase	K07583	tRNA pseudouridine synthase 10	1.3	0.037	0.043	0.021	0.025	0.017
Increase	K07640	Two-component system, OmpR family, sensor histidine kinase CpxA	1.3	0.035	0.040	0.016	0.020	0.012
Decrease	K06145	LacI family transcriptional regulator, gluconate utilization system Gnt-I transcriptional repressor	1.5	0.006	0.007	0.044	0.027	0.033
Decrease	K00338	NADH-quinone oxidoreductase subunit I	1.5	0.028	0.032	0.041	0.029	0.035
Decrease	K07028	Uncharacterized protein	1.4	0.016	0.017	0.030	0.018	0.022
Decrease	K07449	Similar to archaeal holliday junction resolvase and Mrr protein	1.3	0.005	0.005	0.037	0.018	0.022
Decrease	K01751	diaminopropionate ammonia-lyase	1.3	0.025	0.028	0.049	0.040	0.044
Decrease	K01277	dipeptidyl-peptidase III	1.3	0.033	0.037	0.053	0.049	0.058
Decrease	K01218	mannan endo-1,4-beta-mannosidase	1.3	0.005	0.005	0.068	0.054	0.058
Decrease	K01225	cellulose 1,4-beta-cellobiosidase	1.3	0.007	0.007	0.017	0.006	0.010

Data are presented as the medians. Data show the ortholog that increased and decreased in AOM/DSS group (Male_AOM/DSS). LDA effect size, Linear discriminant analysis (LDA) effect size; FDR, false discovery rate; Con, control; AOM, azoxymethane; DSS, dextran sulfate sodium salt; E2, 17β-Estradiol.

## Discussion

A direct interaction between gut microbiota, sex hormones, and the incidence of diseases has been determined based on the data obtained from many studies using animal models, thus suggesting that the potential differences in the composition of the gut microbiota between different sex^34^. The aim of previous studies was to investigate whether tumorigenesis is reduced by estrogen supplementation in AOM/DSS-induced CRC male or OVX female mice model^31,35^. We reported that E2 inhibited the initiation of CRC development by upregulating Nrf2 in AOM/DSS-treated male ICR mice^31^. Furthermore, AOM/DSS-induced proximal colon cancer after ovariectomy was reduced by E2 supplementation^35^. The stool used in this study was taken from the previous experimental sets^31,35^ and the current study was conducted as a secondary outcome. In this study, we investigated whether the composition of the gut microbiota is altered differently between sex and its correlation with the presence of estrogen and development of CRC. The results of this study demonstrated that estrogen could alter the gut microbiota in ICR mice, particularly in male mice treated with AOM/DSS upon decreasing the F/B ratio.

Members of Firmicutes and Bacteroidetes were the most common bacterial phyla constituting the human microbiome. Therefore, the F/B ratio is used as a representative index to compare between different microbial communities^36^. Many studies have supported the idea that a low F/B ratio signifies a healthy condition^36,37^. Additionally, a corresponding decrease in the F/B ratio was observed in patients with CRC, which could be an important marker for intestinal dysbiosis^38,39^. A higher F/B ratio at a BMI of 33 was observed in men compared to that in women^40^. Contrarily, a significantly lower F/B ratio was observed in men than that in women in the group with BMI > 33^40^. Furthermore, evidence from many studies has suggested that the F/B ratio was higher in normal range women than in men. Varied meanings of the F/B ratio between different investigations have been reported. We have previously reported that colitis associated colon carcinogenesis in AOM/DSS-treated mice model was induced more severely in male mice than female by way of the inflammatory mediators such as IL-1β and MPO^30^. Furthermore, we found that E2 inhibited the initiation of CRC by up-regulating nuclear factor erythroid 2-related factor 2 (Nrf2), a transcriptional factor-related pathways in the AOM/DSS-treated male ICR mice^31^. The stool from the previous experimental sets^31,35^ was taken and the current study was conducted. The target protein expression such as IL-1β and MPO was strongly affected by estrogen in acute treatment condition. However, the modest changes on gut microorganisms observed in this study may be originated from too short of intervention, which is a limitation of this study. In the current study, no significant difference in the F/B ratio was observed in the Group 1 samples based on the differences in sex and the presence or absence of estrogen. Interestingly, the F/B ratio was significantly decreased upon estrogen supplementation in the male AOM/DSS group relative to Male_AOM/DSS group. These results have supported that estrogen treatment in CRC models could decrease intestinal dysbiosis by decreasing the F/B ratio.

Different from gender which is related to the social and cultural role of human, sex is biologically determined based on the physiology of the reproductive systems and functions derived from the types of chromosomes or hormones. The terms of ‘male’ and ‘female’ are used to describe the sex of human participants or other sex-related factors^41^. Despite differences in sex, most preclinical studies have adopted the use of male animals, and a few have not mentioned the sex of the animal used^42^. In the present study, we used both male and female mice and investigated the impact of sex hormones, particularly, the female sex hormone ‘estrogen’, on changes in the composition of the gut microbiota. Furthermore, the female mice were subjected to an OVX operation to determine the effect of endogenous estrogen. The results derived from these experiments signify the importance of our research. According to an animal study reported by Org et al., independent analysis of the gut microbiota using 89 different inbred strains of mice was conducted and clear differences in the composition of the gut microbiota and diversity between the sex within each strain was observed^25^. Particularly, obvious differences in the results between sex were observed in the C57BL/6J and C3H/HeJ strains^25^. In the total animal experimental cohort, the members of the phyla Actinobacteria and Tenericutes were more abundant in the male mice than in female mice^25^. In our present study, abundance of the phyla Tenericutes was not correlated with sex. However, its proportion was higher in male mice than in male mice supplemented with E2. Furthermore, at the species level, the abundance of *Bacteroides thetaiotaomicron* was found to be increased in male mice relative to those supplemented with E2, which was similar to the results observed in a previous study conducted in 2008 involving Chinese family members^43^. In another large cohort study, it was determined that females in the Netherlands had a higher proportion of *Akkermansia muciniphila* even after correcting all potential risk factors such as diet, lifestyle, and medication for the normal health^27^. In a Japanese study, significantly higher levels of members belonging to the genus *Akkermansia* were observed in the females than in males^44^ and higher levels of *Akkermansia muciniphila* were identified in C57BL/6 background female relative to male mice^45^. Additionally, an increased abundance of *Clostridium bolteae* was observed in humans after bilateral ovariectomy^27^. Unlike the compositional change of the gut microbiota in humans, which is dependent on sex and the presence of estrogen, in the present study, the abundance of *Akkermansia muciniphila* did not increase in female mice compared to that of the male mice. Contrarily, the abundance of *Akkermansia muciniphila* was found to be increased in OVX female mice compared with that of the intact female mice, which was consistent with the results presented in a previous report by Choi et al.^46^ *Akkermansia muciniphila* is a mucin-degrading bacteria that can convert mucin to short-chain fatty acids^47^. In addition to its role as a commensal bacteria, it has been reported to be involved in pro-inflammatory pathways and the activation of chemotaxis and the complement cascade^48^. Furthermore, it has been reported that the organism had exacerbated an intestinal inflammation in mice infected with *Salmonella typhimurium* by disturbing host mucus homeostasis^49^. Although no change in abundance of *Akkermansia muciniphila* based on sex was observed, its increase in the OVX female group suggested that mucin homeostasis was disrupted due to estrogen removal.

The etiology of CRC development has been investigated in human patients and experimental animal models. It was concluded that the genetic (e.g. diabetes^50^ and obesity^51^) and dietary risk factors (e.g. high fat diet^52^ and processed and red meat^53^) are involved in its development. However, according to a recent review article, it has been suggested that the intestinal microbial community may also be an important contributing factor in the initiation and development of CRC^54^. The intestinal microflora of DSS-treated C57BL/6 background mice has been characterized by a loss in microbial diversity and changes taking place in the bacterial composition, which included a decrease in the number of bacteria from the *Lactobacillus* group and an increase Enterobacteriaceae, *Akkermansia* and *Desulfovibrio*^16^. Furthermore, it is well known that *Fusobacterium nucleatum* is highly associated with CRC development^55^. In the present study, *Fusobacterium nucleatum* was not identified in the experimental CRC group. Contrary to the results generated in the study involving C57BL/6 mice^16^, at the species level, a higher abundance of *Lactobacillus reuteri* was observed in AOM/DSS-induced CRC mice compared to that present in intact male mice. Contrarily, a higher abundance of the species *Lactobacillus murinus* was observed in AOM/DSS-treated male mice supplemented with E2 compared with that of the AOM/DSS-treated male mice. In the present study, *Lactobacillus reuteri* is a well-studied probiotic bacterium capable of colonizing in the bodies of many mammals^56^. In humans, the species has been detected from different sites of the body, including the gastrointestinal tract, urinary tract, skin, and breast milk^56^. *Lactobacillus reuteri* can produce antimicrobial compounds such as organic acids, ethanol, and reuterin. Due to its antimicrobial activity, *Lactobacillus reuteri* is able to inhibit the colonization of pathogenic microbes and remodel the composition of commensal microbiota in the host^56^. Our data suggest that *Lactobacillus reuteri* and *Lactobacillus murinus* may differently act during CRC progression.

It would have been insightful if we could simultaneously verify the effects of testosterone and estrogen, which is a limitation of this study. Testosterone, which is the primary male sex hormone, plays a pivotal role in the development of male reproductive organs such as the testes and prostate and promotes the development of secondary sexual characteristics such as muscle strength and bone mass. Additionally, testosterone is involved in the health and wellbeing of the individual^57^. Therefore, to support the limitation of this study, we are conducting experiments in which animals are subjected to orchiectomy. To confirm the impact of testosterone on the gut microbiota, which consequently is impacted by differences in sex and CRC development, a study involving orchiectomy study is needed. Unfortunately, it was difficult to find models with consistent gut microbiota among sex and affected by sex hormones. However, few were available, which could be identified based on the functional profiles of microbial communities in mice of different sex and in which, development of CRC was impacted due to the presence or absence of estrogen. Currently, we are conducting experiments to analyze gut microbiota according to sex differences in another strain, C57BL/6 mice. We have not verified sex differences in gut microorganisms in the AOM/DSS group, which is a limitation of this study. This is also currently under study in the C57BL/6 strain. In addition, it was regrettable that there was no consideration for progesterone therapy and that the estrogen supplementation group was not added after ovariectomy.

In conclusion, we can state that estrogen altered the gut microbiota in ICR mice, particularly in males treated with AOM/DSS, by decreasing the F/B ratio. This suggests another possibility that estrogen could cause changes in the gut microbiota, thus reducing the risk of developing CRC.

## Methods

### Animals and experimental design

Three and four-week-old female and male ICR mice (Orient Co., Ltd., Seoul, Korea) were housed in cages at 23 °C and subjected to a 12/12-h light/dark cycle under specific pathogen-free conditions. The experimental design is presented in Fig. 1. The experiments were conducted on models distinguished into two categories, which were as follows: (1) sex and OVX as Group 1, and (2) colon cancer in males as Group 2. Group 1 was further divided into 4 subgroups, which were as follows: (1) normal male control mice (n = 5), (2) E2 supplemented male mice (n = 12), (3) normal female control mice (n = 11), and (4) ovariectomized (OVX) female mice (n = 7) (Fig. 1a). Similarly, group 2 was divided into 3 subgroups, which were as follows: (1) normal male control mice (n = 5), (2) AOM/DSS-treated male mice (n = 12), and (3) AOM/DSS-treated male mice supplemented with E2 (n = 12) (Fig. 1b).

After 1 week of acclimatization, the 4-week-old female mice were subjected to ovariectomy in order to create an animal model that could mimic the physiology during menopause through the elimination of endogenous female sex hormones. The normal female control mice were subjected to a sham operation. E2 (10 mg/kg; Sigma-Aldrich, E8876) was dissolved in olive oil, and Male_E2 mice group was received a daily intraperitoneal injection of E2 for 7 days. Except Male_E2 group, others were injected olive oil as a vehicle.

To induce colitis-associated CRC, 5-week-old male mice were injected intraperitoneally with AOM (10 mg/kg; Sigma-Aldrich, St. Louis, MO, USA) and 2.5% (w/v) DSS (MP Biomedicals, Aurora, OH, USA) was administered through their drinking water for 7 days starting 1 week following the injection of AOM^58^. E2-treated mice received an intraperitoneal injection of E2 dissolved in olive oil daily for 7 days. The injections were administered at the time of DSS consumption. Their feces were freshly collected from 59 individual male and female mice in fifteen separate cages. All fecal samples were immediately frozen in liquid nitrogen and stored at − 80 °C. The animals were euthanized by CO_2_ asphyxiation (Fig. 1). The stool from the previous experimental sets^31,35^ was taken and the current study was conducted. Mice were randomly divided from shipment group. All mice were housed in the same room in filter top cages, with three to five mice per cage. Mice were marked so that individual mice could be followed for the duration of the experiments. All animal experimental procedures were approved by the Institutional Animal Care and Use Committee (IACUC) of the Seoul National University Bundang Hospital (BA1310-139/091-01). The procedures were carried out in accordance with the ARRIVE (Animals in Research: Reporting of in Vivo Experiments) guideline.

### DNA extraction and metagenome sequencing of the 16S rRNA gene

Genomic DNA was extracted from the frozen fecal samples using a QIAamp DNA Stool Mini Kit (Qiagen, Valencia, CA, United States) according to the manufacturer’s recommendations. PCR amplification was carried out using the primers targeting the V3 to V4 regions of the 16S rRNA gene of the extracted DNA to conduct metagenome sequencing, and were as follows^59^: 341F (5′-TCGTCGGCAGCGTCAGATGTGTATAAGAGACAGCCTACGGGNGGCWGCAG-3′) and 805R (5′-GTCTCGTGGGCTCGGAGATGTGTATAAGAGACAGGACTACHVGGGTATCTAATCC-3′). Next procedures were performed as described previously^59^. Briefly, the identity of the PCR products was confirmed using electrophoresis. They were purified using a QIAquick PCR Purification Kit (Qiagen, Valencia, CA, USA). The purified PCR products were tagged with Illumina indices and adapters from a Nextera XT Index Kit (Illumina, San Diego, CA, USA). Short DNA fragments were eliminated using a FavorPrep Gel/PCR Purification Kit (Favorgen, Taiwan). The PCR amplicons were quantified using a Quant-iT PicoGreen dsDNA Assay Kit (Thermo Fisher Scientific, Wilmington, DE, USA). After pooling the DNA (300 ng per sample), the PCR products were purified using a FavorPrep Gel/PCR Purification Kit (Favorgen, Taiwan). The quality assessment for confirming the integrity and product size of the DNA was conducted using a Bioanalyzer 2,100 (Agilent, Palo Alto, CA, USA) using a DNA 7,500 chip at ChunLab, Inc. (Seoul, South Korea). Metagenome sequencing was carried out using the Illumina MiSeq platform at ChunLab, Inc. (Seoul, South Korea).

The raw reads were processed by first checking the quality wherein low quality (< Q25) reads were filtered out using a Trimmomatic 0.32^60^. The paired-end sequence data was merged using PANDAseq^61^. The primer sequences were then trimmed using an inhouse program of the ChunLab, Inc. (Seoul, South Korea) with a similarity cut off of 0.8. Non-specific amplicons, which did not encode 16S rRNA, were identified by using the HMMER program hmmsearch based on the 16S rRNA profiles^62^. The sequences were denoised using a DUDE-Seq^63^, and non-redundant reads were extracted through UCLUST-clustering^64^. The EzBioCloud database was utilized for performing the taxonomic assignment using USEARCH (8.1.1861_i86linux32)^64^ followed by more precise pairwise alignment^65^. UCHIME^66^ and the non-chimeric 16S rRNA database from EzBioCloud were used to detect the chimaeras for reads containing a best hit similarity rate of less than 97%. The sequence data was then clustered using CD-HIT^67^ and UCLUST^64^.

Rarefaction for the obtained OTUs was calculated using the BIOiPLUG (ChunLab, Inc.). The reads were normalized to 7,525 to perform the analyses. The microbial diversity (Observed OTU count, Chao1, Shannon, and Simpson indices) was examined using BIOiPLUG. To visualize differences in the samples, PCoA was assessed using generalized UniFrac. The clustering of samples was explained based on the values of the principal coordinate (PC). Additionally, an Unweighted Pair Group Method with Arithmetic mean (UPGMA) tree was created using BIOiPLUG (ChunLab, Inc.). The beta diversity distances were calculated using generalized UniFrac. The significance of the separation between the groups was calculated by permutational multivariate analysis of variance (PERMANOVA) test. Taxonomic summary bar charts were created to determine the OTU abundance ratio (%) at the phylum and family levels using GraphPad Prism (version 5.01). The enterotype classification of the gut microbiota at the genus level was calculated using the R package “clusterSim”. The optimal cluster number was determined by maximizing the value of the Calinski-Harabasz (CH) index^68^.

### Biomarker discovery through the linear discriminant analysis (LDA) effect size (LEfSe)

Based on the relative taxonomic abundances, the taxonomic biomarker discovery and related statistical significance were assessed by the LEfSe method^69^. The criteria for conducting the LEfSe analysis were as follows: (1) an alpha value for the factorial Kruskal–Wallis H test between assigned taxa compared to that of the groups with less than 0.05, (2) an alpha value for the pairwise Wilcoxon test among the taxonomic compositions of less than 0.05, (3) a threshold of the logarithmic LDA score for discriminative features less than 2.0, and (4) a multi-class analysis set as all-against-all. After this process, we went further to simplify the LEfSe plot. (5) overlapped bacteria selection, (6) search and classify the bacterial characteristics with reference to reported papers: commensal bacteria, opportunistic pathogens, not characterized, and (7) elimination of non-overlapped bacteria only at ‘not characterized’ bacteria. In LEfSe plot, all of commensal bacteria and opportunistic pathogens and only overlapped ‘not characterized’ bacteria are included.

To predict the functional biomarker profiles of bacterial communities, we conducted their phylogenetic investigations through reconstructing of unobserved states (PICRUSt)^70^ based on the Kyoto Encyclopedia of Genes and Genomes (KEGG) database (www.kegg.jp/kegg/kegg1.html). Discovery of the functional biomarker and associated statistical significance were assessed based on the LEfSe.

### Statistical analysis

Statistical calculations except those of the pyrosequencing data were performed using PASW Statistics version 18.0.0 (SPSS Inc., 2009, Chicago, IL, USA). The groups were compared based on the results of the Kruskal–Wallis *H* test. Then, the two groups were compared using the Mann–Whitney *U* test (also known as the Wilcoxon rank sum test), and the *p*-value < 0.05 was considered to be significant. For the adjustment of multiple comparisons, corrected q-values of the false discovery rate (FDR) were calculated for significance less than 5%.

## Supplementary information

Supplementary information.

Supplementary dataset 1.

Supplementary dataset 2.

Supplementary dataset 3.

Supplementary dataset 4.

## Data Availability

The raw unprocessed gene datasets of 16S rRNA, which were generated during the current study, are available with the NCBI Sequence Read Archive (SRA Accession Number PRJNA590320), https://www.ncbi.nlm.nih.gov/sra/PRJNA590320. The processed data generated and analyzed during this study has been included in this published article under Supplementary Dataset files.

## References

[1] Siegel RL, Miller KD, Jemal A (2019). Cancer statistics, 2019. CA Cancer J. Clin..

[2] Lakatos PL, Lakatos L (2008). Risk for colorectal cancer in ulcerative colitis: changes, causes and management strategies. World J. Gastroenterol..

[3] Zamor PJ, deLemos AS, Russo MW (2017). Viral hepatitis and hepatocellular carcinoma: etiology and management. J. Gastrointest. Oncol..

[4] Parsonnet J (1991). Helicobacter pylori infection and the risk of gastric carcinoma. N. Engl. J. Med..

[5] Sears CL, Garrett WS (2014). Microbes, microbiota, and colon cancer. Cell Host Microbe.

[6] Zhang S (2019). Fusobacterium nucleatum promotes chemoresistance to 5-fluorouracil by upregulation of BIRC3 expression in colorectal cancer. J. Exp. Clin. Cancer Res..

[7] Claesson MJ (2012). Gut microbiota composition correlates with diet and health in the elderly. Nature.

[8] Martin FP (2008). Probiotic modulation of symbiotic gut microbial-host metabolic interactions in a humanized microbiome mouse model. Mol. Syst. Biol..

[9] Makivuokko H (2012). Association between the ABO blood group and the human intestinal microbiota composition. BMC Microbiol..

[10] Suzuki R, Kohno H, Sugie S, Tanaka T (2004). Sequential observations on the occurrence of preneoplastic and neoplastic lesions in mouse colon treated with azoxymethane and dextran sodium sulfate. Cancer Sci..

[11] Thaker AI, Shaker A, Rao MS, Ciorba MA (2012). Modeling colitis-associated cancer with azoxymethane (AOM) and dextran sulfate sodium (DSS). J. Vis. Exp..

[12] De Robertis M (2011). The AOM/DSS murine model for the study of colon carcinogenesis: from pathways to diagnosis and therapy studies. J. Carcinog..

[13] Wang CZ (2018). Role of intestinal microbiome in American ginseng-mediated colon cancer prevention in high fat diet-fed AOM/DSS mice [corrected]. Clin. Transl. Oncol. Offic. Publ. Fed. Span. Oncol. Soc. Natl. Cancer Inst. Mex..

[14] Zackular JP (2013). The gut microbiome modulates colon tumorigenesis. mBio.

[15] Ibrahim A (2019). Colitis-induced colorectal cancer and intestinal epithelial estrogen receptor beta impact gut microbiota diversity. Int. J. Cancer.

[16] Hakansson A (2015). Immunological alteration and changes of gut microbiota after dextran sulfate sodium (DSS) administration in mice. Clin. Exp. Med..

[17] McCashland TM, Brand R, Lyden E, de Garmo P, Project, C. R (2001). Gender differences in colorectal polyps and tumors. Am. J. Gastroenterol..

[18] Kim SE (2015). Sex- and gender-specific disparities in colorectal cancer risk. World J. Gastroenterol..

[19] Gierisch JM (2013). Oral contraceptive use and risk of breast, cervical, colorectal, and endometrial cancers: a systematic review. Cancer Epidemiol. Biomark. Prev. Publ. Am. Assoc. Cancer Res. Cosponsored Am. Soc. Prev. Oncol..

[20] Chlebowski RT (2004). Estrogen plus progestin and colorectal cancer in postmenopausal women. N. Engl. J. Med..

[21] Jung KW (2014). Cancer statistics in Korea: incidence, mortality, survival, and prevalence in 2011. Cancer Res. Treat..

[22] Sommer F, Backhed F (2013). The gut microbiota–masters of host development and physiology. Nat. Rev. Microbiol..

[23] Tremaroli V, Backhed F (2012). Functional interactions between the gut microbiota and host metabolism. Nature.

[24] Baker JM, Al-Nakkash L, Herbst-Kralovetz MM (2017). Estrogen-gut microbiome axis: physiological and clinical implications. Maturitas.

[25] Org E (2016). Sex differences and hormonal effects on gut microbiota composition in mice. Gut Microbes.

[26] Cox-York KA (2015). Ovariectomy results in differential shifts in gut microbiota in low versus high aerobic capacity rats. Physiol. Rep..

[27] Sinha T (2019). Analysis of 1135 gut metagenomes identifies sex-specific resistome profiles. Gut Microbes.

[28] Flores R (2012). Fecal microbial determinants of fecal and systemic estrogens and estrogen metabolites: a cross-sectional study. J. Transl. Med..

[29] Vieira AT, Castelo PM, Ribeiro DA, Ferreira CM (2017). Influence of oral and gut microbiota in the health of menopausal women. Front. Microbiol..

[30] Lee SM (2016). The effect of sex on the azoxymethane/dextran sulfate sodium-treated mice model of colon cancer. J. Cancer Prev..

[31] Son HJ (2019). Effect of estradiol in an azoxymethane/dextran sulfate sodium-treated mouse model of colorectal cancer: implication for sex difference in colorectal cancer development. Cancer Res. Treat..

[32] Son HJ (2018). 17beta-Estradiol reduces inflammation and modulates antioxidant enzymes in colonic epithelial cells. Korean J. Intern. Med..

[33] Song CH, Kim N, Kim DH, Lee HN, Surh YJ (2019). 17-beta estradiol exerts anti-inflammatory effects through activation of Nrf2 in mouse embryonic fibroblasts. PLoS ONE.

[34] Markle JG (2013). Sex differences in the gut microbiome drive hormone-dependent regulation of autoimmunity. Science.

[35] Song CH (2019). Effects of 17beta-estradiol on colorectal cancer development after azoxymethane/dextran sulfate sodium treatment of ovariectomized mice. Biochem. Pharmacol..

[36] Mariat D (2009). The firmicutes/bacteroidetes ratio of the human microbiota changes with age. BMC Microbiol..

[37] Turnbaugh PJ (2006). An obesity-associated gut microbiome with increased capacity for energy harvest. Nature.

[38] Gagniere J (2016). Gut microbiota imbalance and colorectal cancer. World J. Gastroenterol..

[39] Lucas C, Barnich N, Nguyen HTT (2017). Microbiota, inflammation and colorectal cancer. Int. J. Mol. Sci..

[40] Haro C (2016). Intestinal microbiota is influenced by gender and body mass index. PLoS ONE.

[41] Kim YS, Unno T, Kim BY, Park MS (2019). Sex differences in gut microbiota. World J. Mens Health.

[42] Jahng J, Kim YS (2017). Why should we contemplate on gender difference in functional gastrointestinal disorders?. J. Neurogastroenterol. Motil..

[43] Li M (2008). Symbiotic gut microbes modulate human metabolic phenotypes. Proc. Natl. Acad .Sci. USA.

[44] Gao X (2018). Body mass index differences in the gut microbiota are gender specific. Fronti. Microbiol..

[45] Kaliannan K (2018). Estrogen-mediated gut microbiome alterations influence sexual dimorphism in metabolic syndrome in mice. Microbiome.

[46] Choi S, Hwang YJ, Shin MJ, Yi H (2017). Difference in the gut microbiome between ovariectomy-induced obesity and diet-induced obesity. J. Microbiol. Biotechnol..

[47] Derrien M, Vaughan EE, Plugge CM, de Vos WM (2004). *Akkermansia muciniphila* gen. nov., sp. nov., a human intestinal mucin-degrading bacterium. Int. J. Syst. Evol. Microbiol..

[48] Derrien M (2011). Modulation of mucosal immune response, tolerance, and proliferation in mice colonized by the mucin-degrader Akkermansia muciniphila. Front. Microbiol..

[49] Ganesh BP, Klopfleisch R, Loh G, Blaut M (2013). Commensal akkermansia muciniphila exacerbates gut inflammation in salmonella typhimurium-infected gnotobiotic mice. PLoS ONE.

[50] Larsson SC, Orsini N, Wolk A (2005). Diabetes mellitus and risk of colorectal cancer: a meta-analysis. J. Natl Cancer Inst..

[51] Bardou M, Barkun AN, Martel M (2013). Obesity and colorectal cancer. Gut.

[52] Norat T (2005). Meat, fish, and colorectal cancer risk: the European prospective investigation into cancer and nutrition. J. Natl Cancer Inst..

[53] Larsson SC, Wolk A (2006). Meat consumption and risk of colorectal cancer: a meta-analysis of prospective studies. Int. J. Cancer.

[54] Gao R, Gao Z, Huang L, Qin H (2017). Gut microbiota and colorectal cancer. Eur. J. Clin. Microbiol. Infect. Dis. Off. Publ. Eur. Soc. Clin. Microbiol..

[55] Shang FM, Liu HL (2018). Fusobacterium nucleatum and colorectal cancer: a review. World J. Gastrointest. Oncol..

[56] Mu Q, Tavella VJ, Luo XM (2018). Role of lactobacillus reuteri in human health and diseases. Front. Microbiol..

[57] Bassil N, Alkaade S, Morley JE (2009). The benefits and risks of testosterone replacement therapy: a review. Ther. Clin. Risk Manag..

[58] Yum HW (2013). Oligonol inhibits dextran sulfate sodium-induced colitis and colonic adenoma formation in mice. Antioxid. Redox Signal..

[59] Lee SM (2019). Gut microbiota and butyrate level changes associated with the long-term administration of proton pump inhibitors to old rats. Sci. Rep..

[60] Bolger AM, Lohse M, Usadel B (2014). Trimmomatic: a flexible trimmer for Illumina sequence data. Bioinformatics (Oxford, England).

[61] Masella AP, Bartram AK, Truszkowski JM, Brown DG, Neufeld JD (2012). PANDAseq: paired-end assembler for illumina sequences. BMC Bioinform..

[62] Eddy SR (2011). Accelerated profile HMM searches. PLoS Comput. Biol..

[63] Lee B, Moon T, Yoon S (2017). DUDE-Seq: fast, flexible, and robust denoising for targeted amplicon sequencing. PLoS ONE.

[64] Edgar RC (2010). Search and clustering orders of magnitude faster than BLAST. Bioinformatics (Oxford, England).

[65] Myers EW, Miller W (1988). Optimal alignments in linear space. Comput. Appl. Biosci. CABIOS.

[66] Edgar RC, Haas BJ, Clemente JC, Quince C, Knight R (2011). UCHIME improves sensitivity and speed of chimera detection. Bioinformatics (Oxford, England).

[67] Fu L, Niu B, Zhu Z, Wu S, Li W (2012). CD-HIT: accelerated for clustering the next-generation sequencing data. Bioinformatics (Oxford, England).

[68] Liu, Y., Li, Z., Xiong, H., Gao, X. & Wu, J. In *2010 IEEE International Conference on Data Mining*, 911–916.

[69] Segata N (2011). Metagenomic biomarker discovery and explanation. Genome Biol.

[70] Langille MG (2013). Predictive functional profiling of microbial communities using 16S rRNA marker gene sequences. Nat. Biotechnol..

