# Supplementary material for: 17β-Estradiol supplementation changes gut microbiota diversity in intact and colorectal cancer-induced ICR male mice
Source: Sci Rep. 2020 Jul 23;10:12283. doi: 10.1038/s41598-020-69112-w (PMC7378548; doi:10.1038/s41598-020-69112-w)
Supplement: Supplementary file 1 — Supplementary information. [file 41598_2020_69112_MOESM1_ESM.pdf]

Supplementary Information

RE: 17β-Estradiol Supplementation Changes Gut Microbiota Diversity in Intact and Colorectal Cancer-induced ICR Male Mice

Chin-Hee Song[1], Nayoung Kim[1,2]*, Ryoung Hee Nam[1], Soo In Choi[1], Ha-Na Lee[3], and Young-Joon Surh[4]

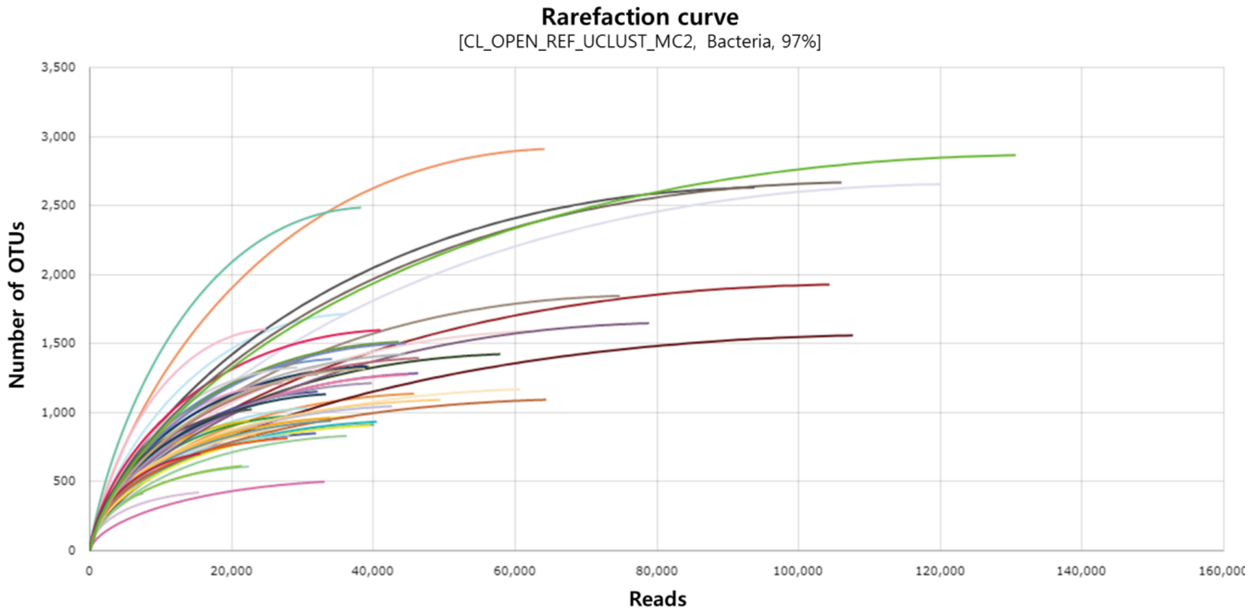

Supplementary Figure 1. Rarefaction curves of the 97% OTUs observed species from total six groups of mice.

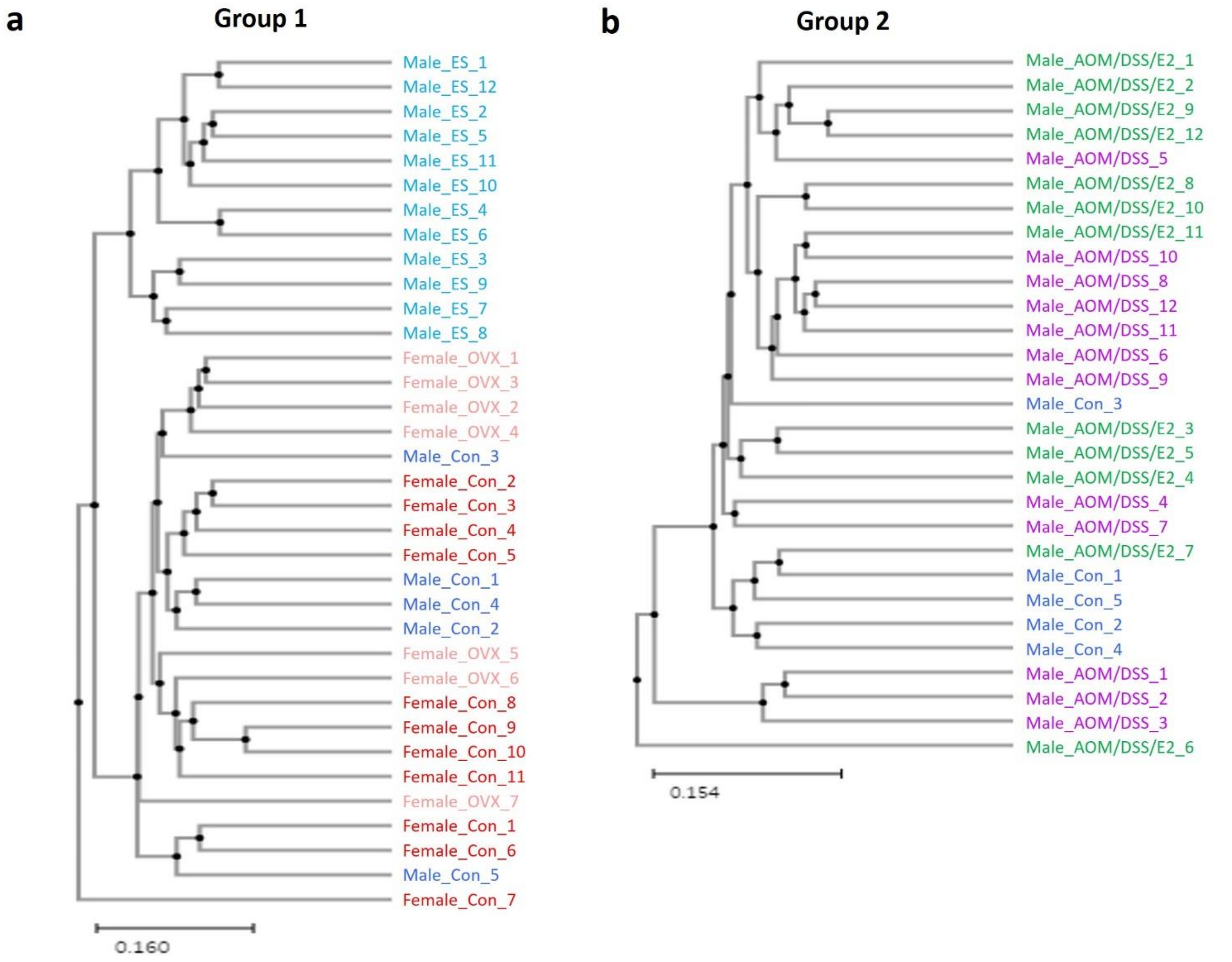

Supplementary Figure 2. Beta diversity of gut microbiota. Unweighted pair-group method with arithmetic mean (UPGMA) tree of Generalized Unifrac distances of the fecal samples from (a) Group1 (Male_Con, Male_E2, Female_Con, and Female_OVX) and (b) Group2 (Male_Con, Male_AOM/DSS, and Male_AOM/DSS/E2). Con, control; E2, 17β-Estradiol; OVX, ovariectomized; AOM, azoxymethane; DSS, dextran sulfate sodium salt.

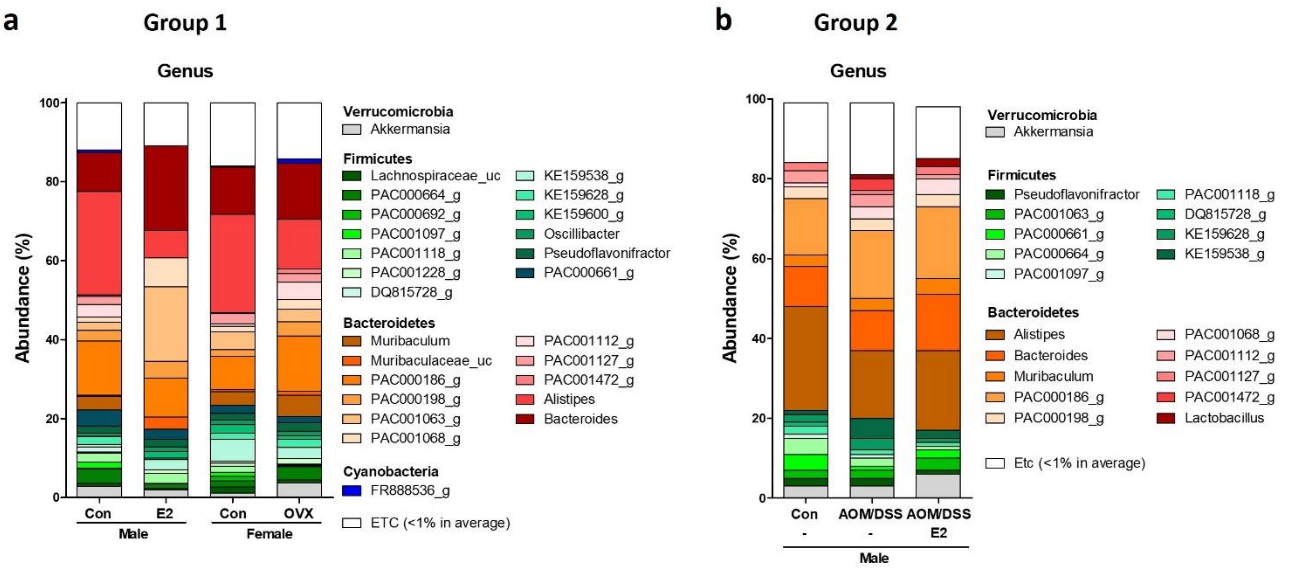

Supplementary Figure 3. Taxonomic composition at the genus level. Microbial composition of the fecal contents from (a) Group1 (Male_Con, Male_E2, Female_Con, and Female_OVX) and (b) Group2 (Male_Con, Male_AOM/DSS, and Male_AOM/DSS/E2). Bar charts show the mean values. Con, control; E2, 17β-Estradiol; OVX, ovariectomized; AOM, azoxymethane; DSS, dextran sulfate sodium salt.

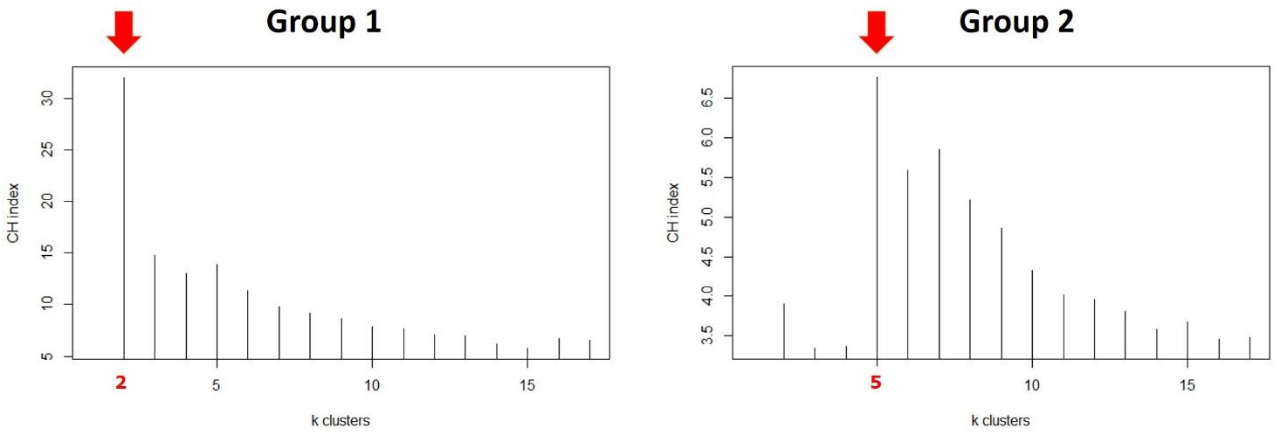

Supplementary Figure 4. Calinski-Harabasz (CH) index for the separation of enterotypes. Enterotypes were determined based on the CH index. (a) Samples from Group1 (Male_Con, Male_E2, Female_Con, and Female_OVX) were separated into two enterotypes based to the highest CH index when k cluster was 2. (b) The CH index of the Group2 samples (Male_Con, Male_AOM/DSS, and Male_AOM/DSS/E2) was highest when k clusters were 5. Con, control; E2, 17β-Estradiol; OVX, ovariectomized; AOM, azoxymethane; DSS, dextran sulfate sodium salt.

Supplementary Table 1. Taxonomic relative abundance of the gut microbiota at the phylum level.

	Abundance (mean, %)			
	Cyanobacteria	Bacteroidetes	Firmicutes	Verrucomicrobia
**Group1**				
Male_con. (n=5)	0.56	66.95	28.88	2.82
Male_E2 (n=12)	0.00	73.04	24.66	1.90
Female_con (n=11)	0.28	61.56	35.98	1.16
Female_ovx (n=7)	1.06	65.56	28.50	3.68
p-value[a]	**<0.001**	0.082	0.206	0.792
p-value[b]	0.258	0.821	0.390	**0.007**
p-value[c]	0.157	0.533	0.462	0.392
p-value[d]	**<0.001**	0.200	0.253	0.054
**Group2**				
Male_con (n=5)	0.56	66.95	28.88	2.82
Male_AOM/DSS (n=12)	0.91	64.84	30.64	2.73
Male_AOM/DSS/E2 (n=12)	0.70	73.21	19.08	6.24
p-value[e]	0.792	0.461	0.527	0.562
p-value[f]	0.644	0.073	0.073	0.686
p-value[g]	0.893	0.076	0.101	0.806

Data are presented as the medians. p-value < 0.05 was considered to be significant and was presented as boldface. Mann–Whitney U-test for comparison difference between independent two groups was performed in a, b, c, e, and f. The difference between Group1 and between Group2 was analysed by Kruskal-Wallis H test in d and g. [a], p-values between Male_Con vs Male_E2; [b], p-values between Female_Con vs Female_OVX; [c], p-values between Male_Con vs Female_Con; [d], p-values between Group1; [e], p-values between Male_Con vs Male_AOM/DSS; [f], p-values between Male_AOM/DSS vs Male_AOM/DSS/E2; [g], p-values between Group2. OTU, operational taxonomic unit; Con, control; E2, 17β-Estradiol; OVX, ovariectomized; AOM, azoxymethane; DSS, dextran sulfate sodium salt.

Supplementary Table 2. Taxonomic relative abundance of the gut microbiota at the family level.

	Abundance (mean, %)							
	FR888536_f	Bacteroidaceae	Rikenellaceae	Muribaculaceae	Christensenellaceae	Ruminococcaceae	Lachnospiraceae	Akkermansiaceae
**Group1**								
Male_con. (n=5)	0.56	9.91	26.25	29.58	0.91	8.30	19.10	2.82
Male_E2 (n=12)	0.00	21.50	6.89	44.26	0.55	6.65	17.25	1.90
Female_con (n=11)	0.28	11.92	25.02	24.12	1.19	6.29	27.06	1.16
Female_ovx (n=7)	1.06	14.32	12.67	38.15	0.24	6.54	21.09	3.68
p-value[a]	**<0.001**	**0.027**	**0.008**	**0.035**	0.114	0.343	0.461	0.792
p-value[b]	0.258	0.821	**0.013**	**0.042**	**0.018**	0.964	0.556	**0.007**
p-value[c]	0.157	0.157	0.865	0.336	0.955	0.062	0.336	0.392
p-value[d]	**<0.001**	**0.017**	**<0.001**	**0.014**	**0.026**	0.464	0.363	0.054
**Group2**								
Male_con (n=5)	0.56	9.91	26.25	29.58	0.91	8.30	19.10	2.82
Male_AOM/DSS (n=12)	0.91	9.95	16.86	37.20	0.34	6.09	23.05	2.73
Male_AOM/DSS/E2 (n=12)	0.70	14.27	20.32	37.47	0.23	5.08	11.68	6.24
p-value[e]	0.792	0.598	0.114	0.206	**0.002**	**0.045**	0.598	0.562
p-value[f]	0.644	0.119	0.225	0.792	0.149	0.356	**0.038**	0.686
p-value[g]	0.893	0.193	0.208	0.449	**0.001**	**0.036**	0.066	0.806

Data are presented as the medians. p-value < 0.05 was considered to be significant and was presented as boldface. Mann–Whitney U-test for comparison difference between independent two groups was performed in a, b, c, e, and f. The difference between Group1 and between Group2 was analysed by Kruskal-Wallis H test in d and g. [a], p-values between Male_Con vs Male_E2; [b], p-values between Female_Con vs Female_OVX; [c], p-values between Male_Con vs Female_Con; [d], p-values between Group1; [e], p-values between Male_Con vs Male_AOM/DSS; [f], p-values between Male_AOM/DSS vs Male_AOM/DSS/E2; [g], p-values between Group2. OTU, operational taxonomic unit; Con, control; E2, 17β-Estradiol; OVX, ovariectomized; AOM, azoxymethane; DSS, dextran sulfate sodium salt.

Supplementary Dataset 1. (The data file is supplied as a separate file: Supplementary_Dataset_1.xlsx.) Operational taxonomic unit (OTU) counts and abundance ratios of each taxon for Group 1. Con, control; E2, 17β-Estradiol; OVX, ovariectomized.

Supplementary Dataset 2. (The data file is supplied as a separate file: Supplementary_Dataset_1.xlsx.) Operational taxonomic unit (OTU) counts and abundance ratios of each taxon for Group 2. Con, control; AOM, azoxymethane; DSS, dextran sulfate sodium salt; E2, 17β-Estradiol.

Supplementary Dataset 3. (The data file is supplied as a separate file: Supplementary_Dataset_3.xlsx.) The p-values and q-values (FDR) of every taxon in gut microbiota of Group 1 and Group 2. P-values were determined with the Kruskal-Wallis H test and q-values were determined by FDR of the p-values by Kruskal-Wallis H test. FDR, false discovery rate; Con, control; E2, 17β-Estradiol; OVX, ovariectomized; AOM, azoxymethane; DSS, dextran sulfate sodium salt.

Supplementary Dataset 4. (The data file is supplied as a separate file: Supplementary_Dataset_4.xlsx.) LDA effect size, p-values, and q-values (FDR) of every taxon in gut microbiota of Group 1 and Group 2 were determined with the LEfSe analysis. LDA, linear discriminant analysis; FDR, false discovery rate; Con, control; E2, 17β-Estradiol; OVX, ovariectomized; AOM, azoxymethane; DSS, dextran sulfate sodium salt.
